# Supplementary material for: Biased genome editing using the local accumulation of DSB repair molecules system
Source: Nat Commun. 2018 Aug 16;9:3270. doi: 10.1038/s41467-018-05773-6 (PMC6095859; doi:10.1038/s41467-018-05773-6)
Supplement: Supplementary file 1 — Supplementary Information [file 41467_2018_5773_MOESM1_ESM.pdf]

# Biased genome editing using the local accumulation of DSB repair molecules system

Shota Nakade, Keiji Mochida, Atsushi Kunii, Kazuki Nakamae, Tomomi Aida, Kohichi Tanaka, Naoaki Sakamoto, Tetsushi Sakuma, and Takashi Yamamoto

**Supplementary Information**

Supplementary Figures 1–21

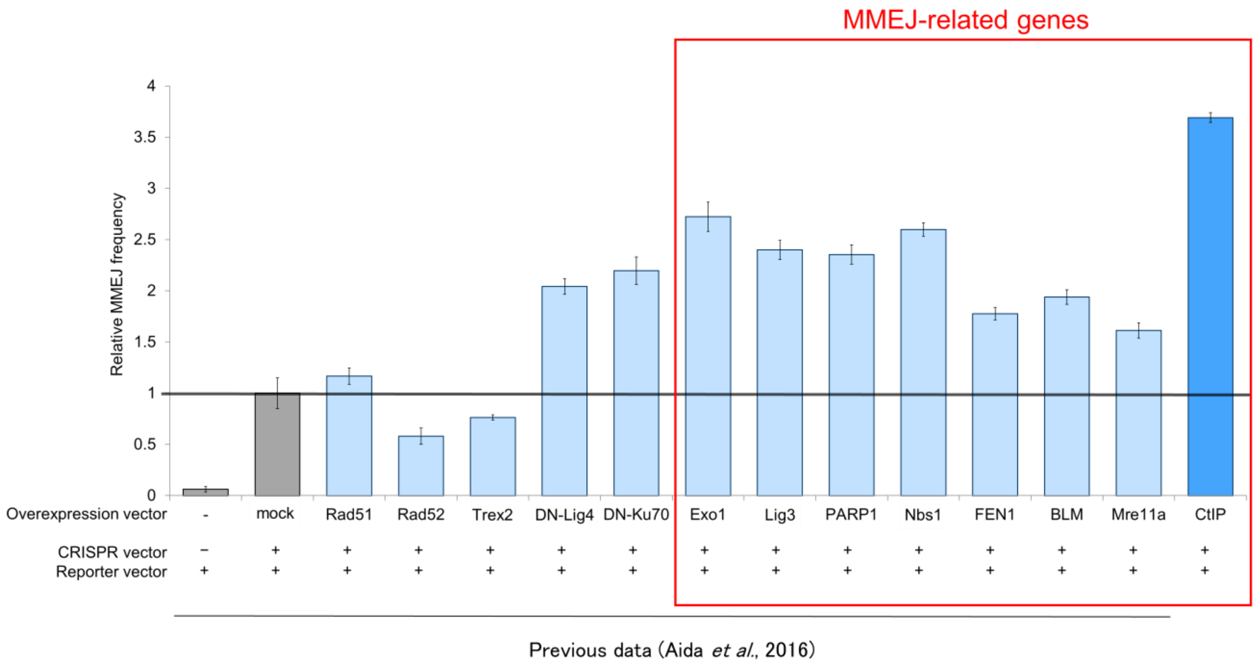


**Supplementary Figure 1. Screening of MMEJ enhancer using the MMEJ-monitoring reporter assay**

Relative MMEJ frequency was determined by the EGFP recovery assay as described previously[8]. The data other than from CtIP transfection were obtained previously[8]. Data are expressed as means ± s.e.m. (n = 4).

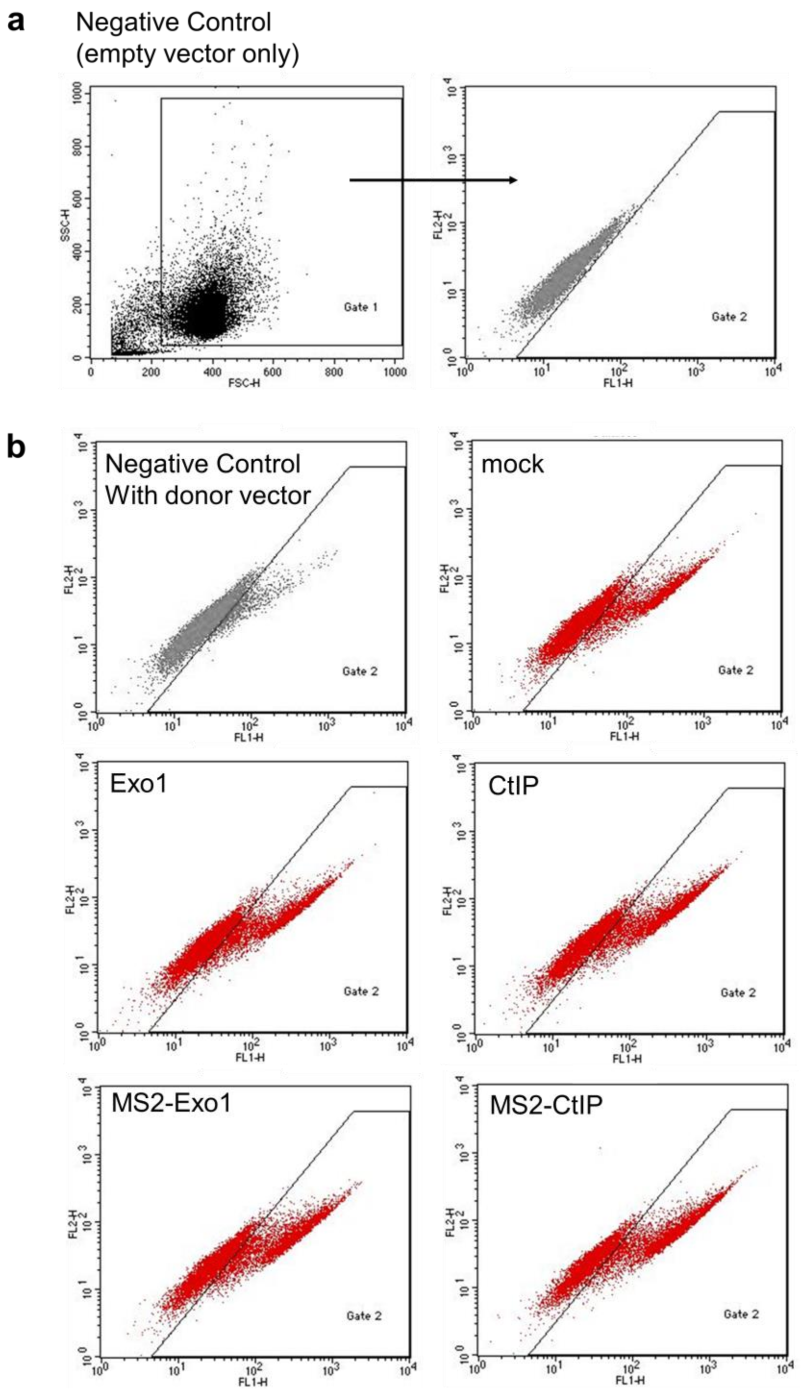


**Supplementary Figure 2. Fluorescence profiles of FACS analysis, related to Figure 1c**

a. Scatter plot of the preliminary FSC/SSC gating (left) and FACS plot of negative control (empty vector only; right).

b. FACS plots of the samples of Figure 1c. FL1-H and FL2-H indicate the intensity of green and red fluorescence, respectively. The cells within the region labeled as Gate 2 were regarded as green fluorescence-positive cells.

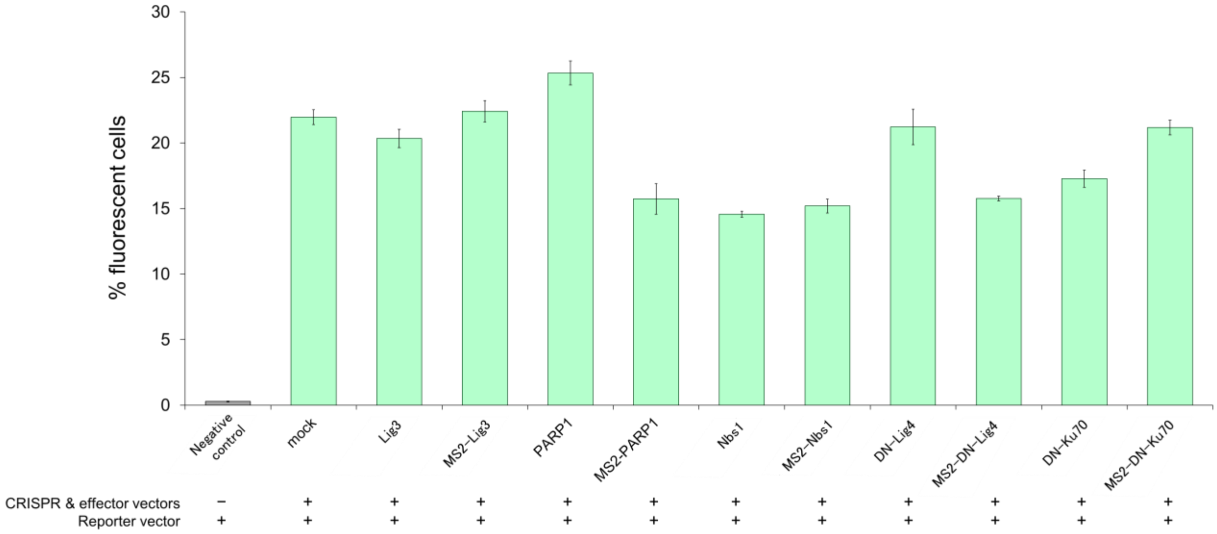


**Supplementary Figure 3. Additional screening of PITCh enhancer, related to Figure 1c**

Knock-in frequency at the *CANX* locus measured by FACS analysis. No significant increase was observed with the LoADing of Lig3, PARP1, Nbs1, dominant-negative Lig4 (DN-Lig4), and dominant-negative Ku70 (DN-Ku70). Data are expressed as means ± s.e.m. (n = 3).

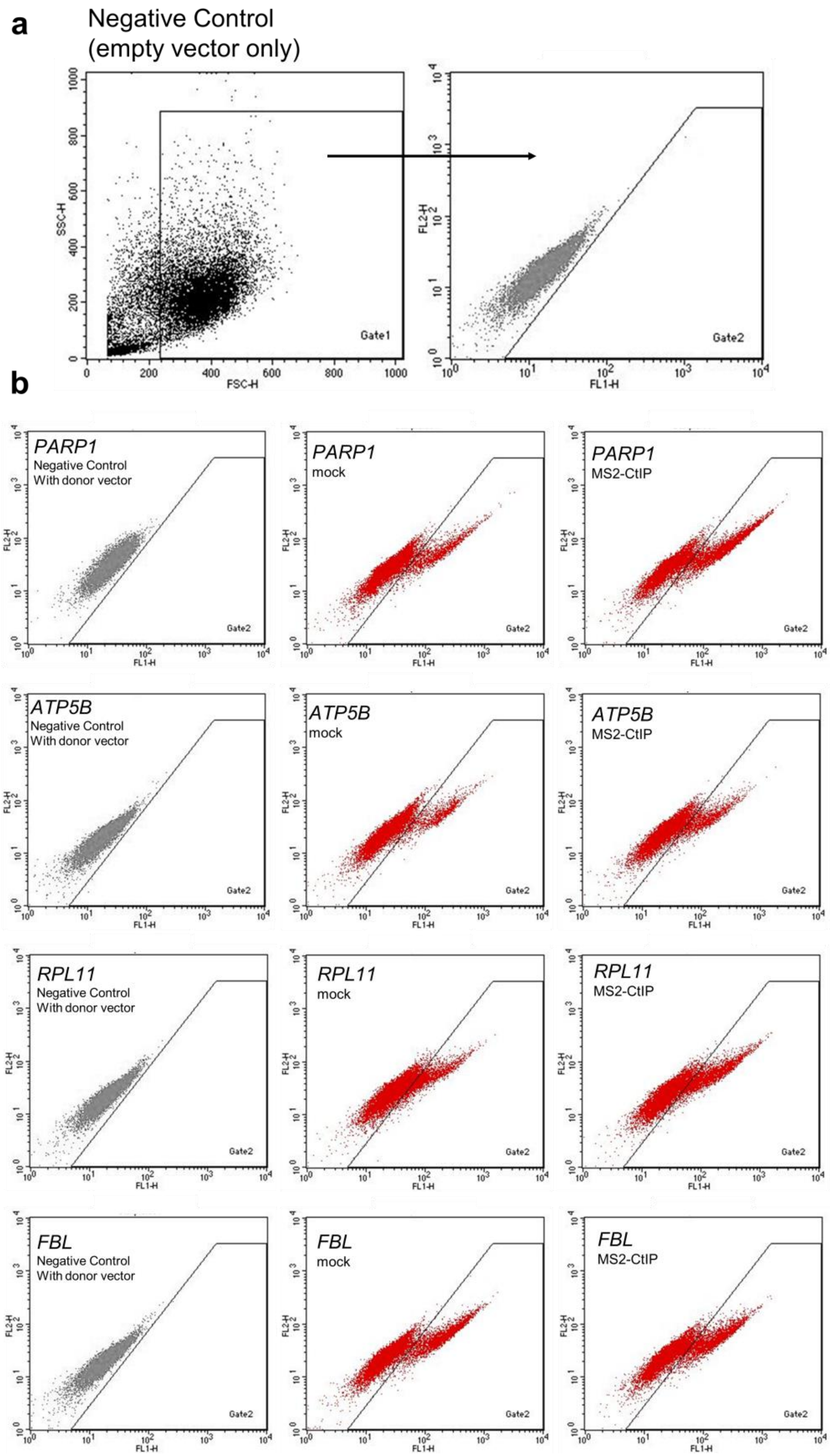


**Supplementary Figure 4. Fluorescence profiles of FACS analysis, related to Figure 1d**

a. Scatter plots of the preliminary FSC/SSC gating (left) and FACS plot of negative control (empty vector only; right).
b. FACS plots of the samples of Figure 1d. FL1-H and FL2-H indicate the intensity of green and red fluorescence, respectively. The cells within the region labeled as Gate 2 were regarded as green fluorescence-positive cells.

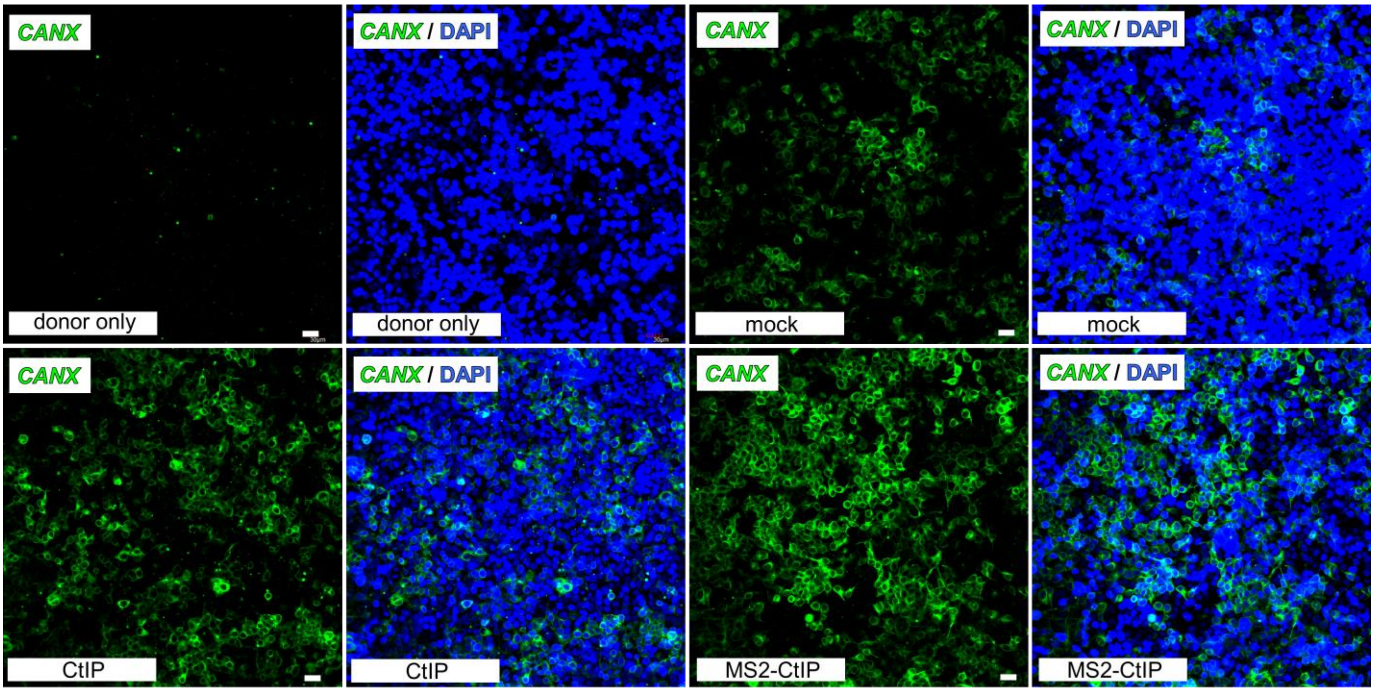


**Supplementary Figure 5. Laser-scanning microscopy images of the cells in which mNeonGreen cDNA was knocked-in at the *CANX* locus with or without MS2-CtIP LoADing**

Fluorescence images of the cells at 72 h post-transfection without any selection are shown. Knock-in cells showed the expected pattern of fluorescence localization, and the frequency of knock-in was highest in the cells transfected with MS2-CtIP, followed by CtIP and mock. Bars, 30 µm.

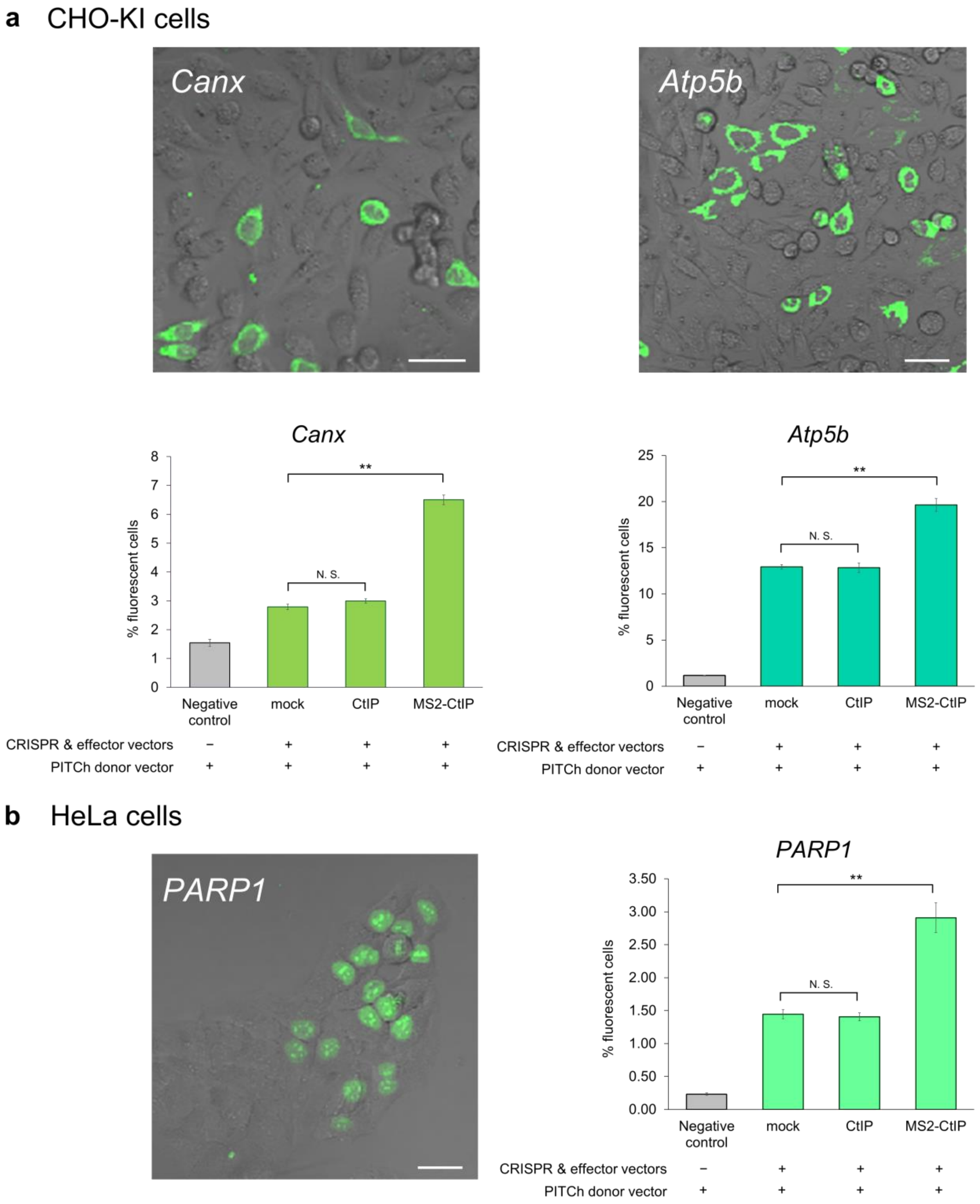


**Supplementary Figure 6. The knock-in-enhancing effect of MS2-CtIP LoADing in CHO-K1 (a) and HeLa (b) cells**

Fluorescence images and percentages of fluorescent cells calculated by FACS analysis are shown. Bars, 30 μm. FACS data are expressed as means ± s.e.m. (n = 3). **P < 0.01 (Student's t-test). N.S., not significant.

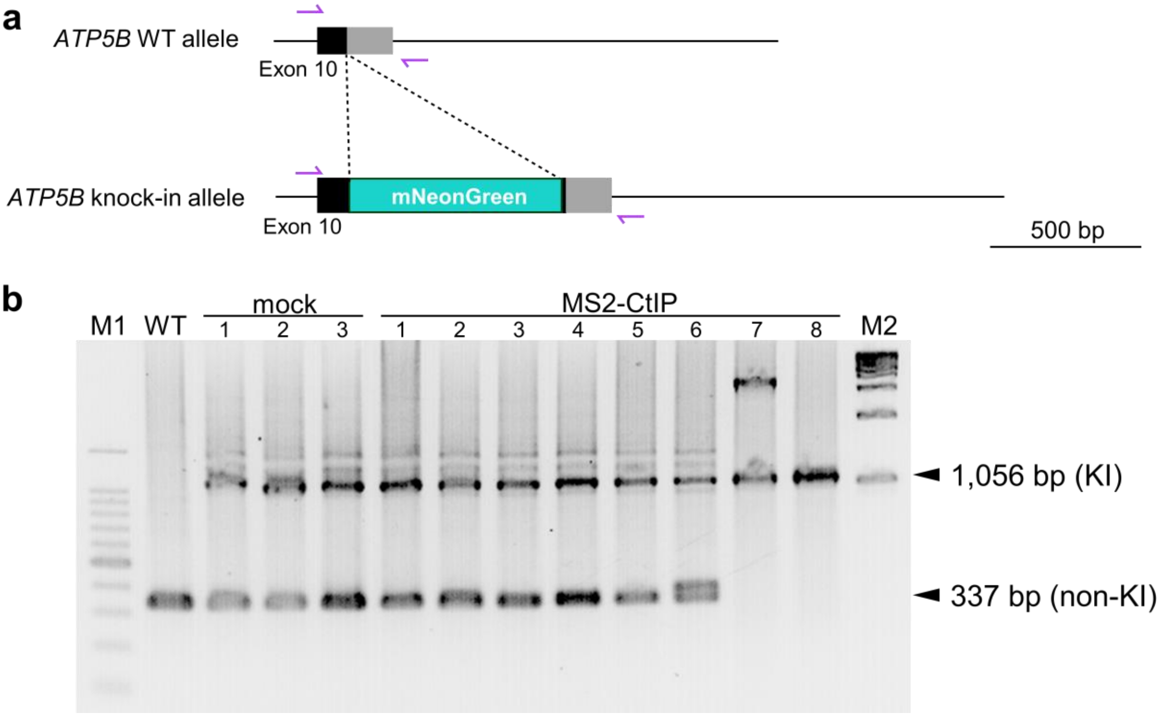


**Supplementary Figure 7. Out-out PCR analysis of knock-in clones established with the conventional PITCh and the LoADed PITCh systems.**

a. Schematic of wild-type (WT) and knock-in alleles. Arrows indicate the positions of primers.

b. An inverted agarose gel image of the out-out PCR products. The numbers indicate clone IDs. The expected sizes of knock-in (KI) and non-knock-in (non-KI) bands are shown by arrowheads. M1, 100-bp ladder marker. M2, 1-kb ladder marker.

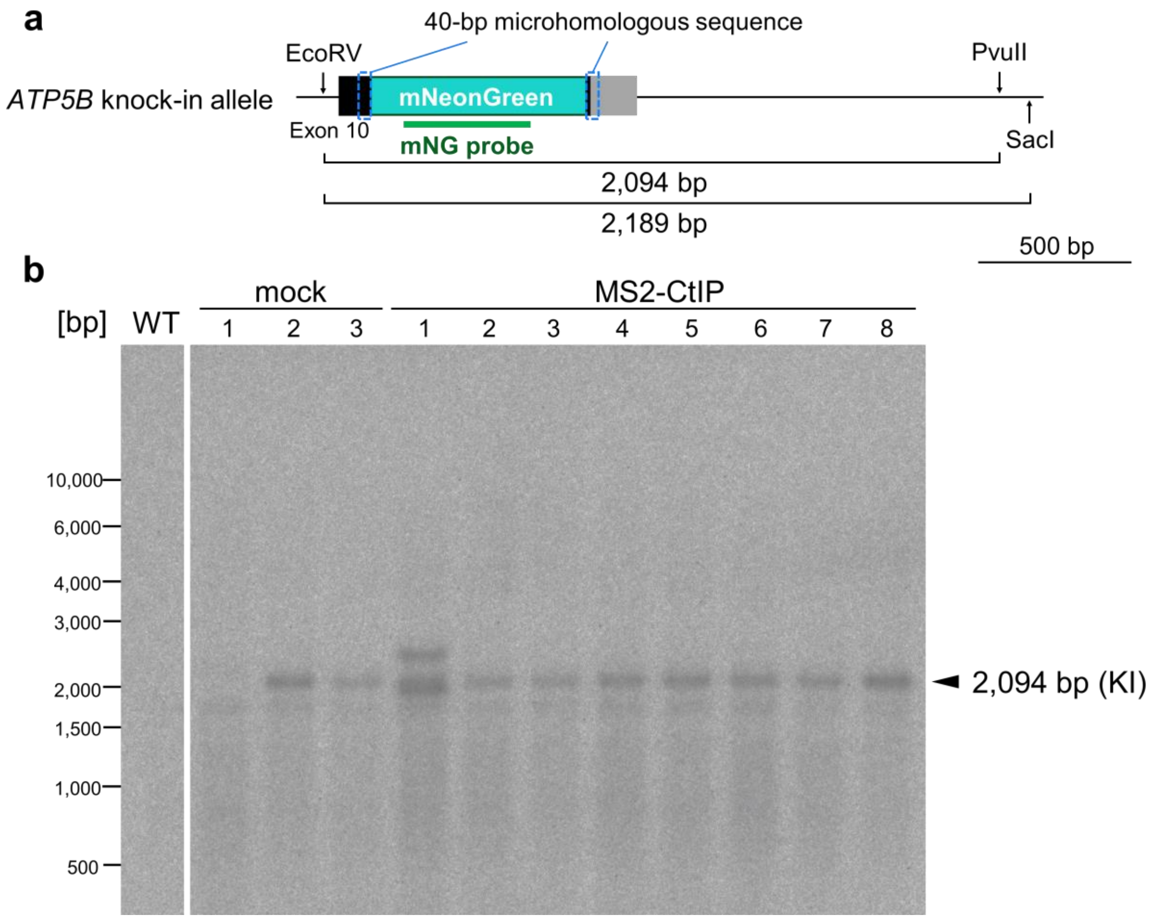


**Supplementary Figure 8. Southern blot analysis.**

a. Schematic of knock-in allele with the annotations of the positions of designed probe, microhomologies, and restriction sites, as well as the expected sizes of detectable DNA fragments.
b. The radio-isotope image of Southern blot analysis. The numbers indicate clone IDs. The expected size of knock-in (KI) band is shown by an arrowhead. WT, wild-type.

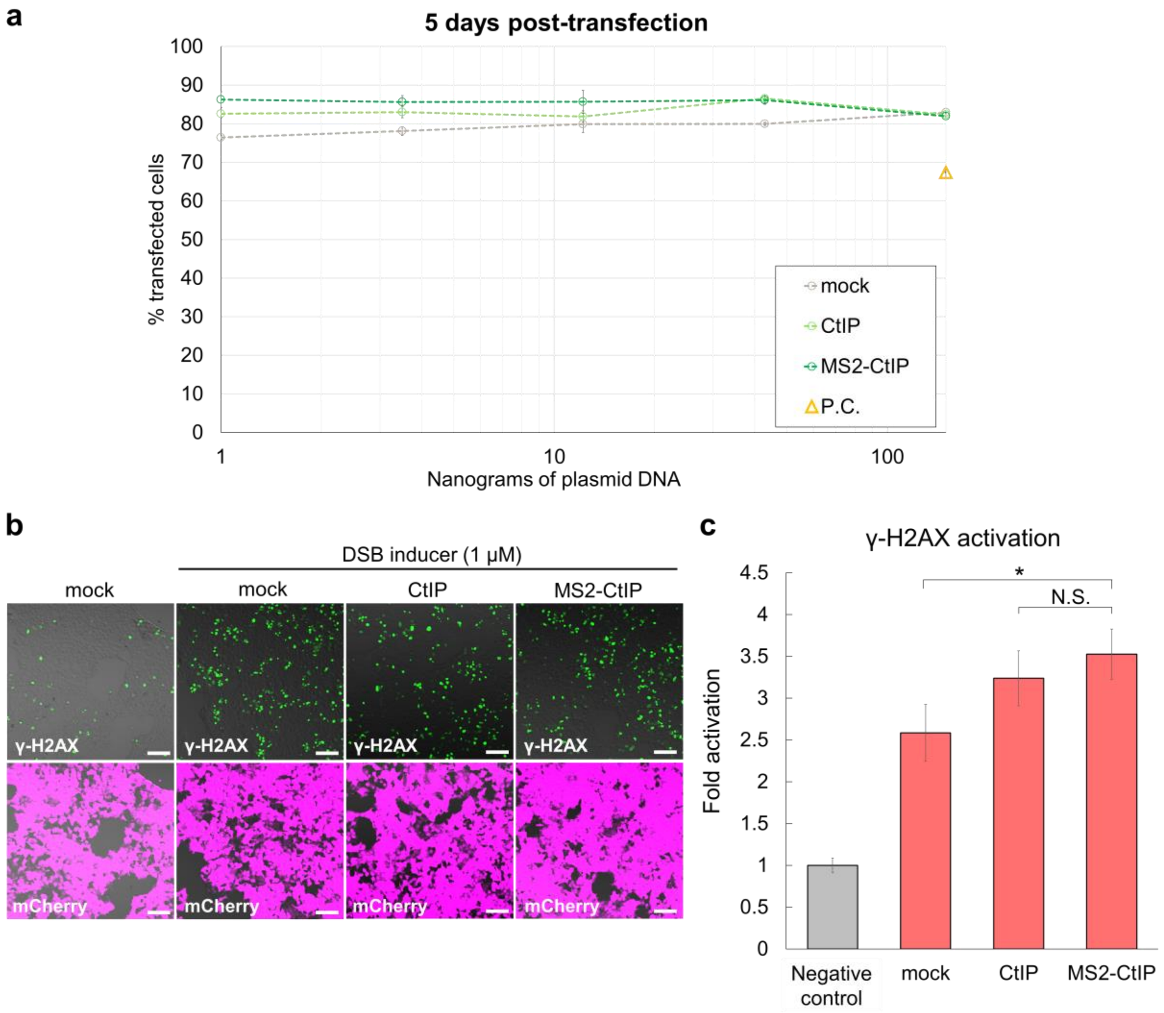


**Supplementary Figure 9. Cell viability assay and γ-H2AX immunostaining.**

a. The percentages of cell survival were determined at 5 days post-transfection. The data of mock vector-, CtIP vector-, MS2-CtIP vector-, and ZFN vector-transfected samples are shown. ZFN was used to validate whether the cell toxicity can be measured in our experiment, because it has been reported to be toxic in human cells. Data are expressed as means ± s.e.m. (n = 3). P.C., positive control ZFN.

b. Laser-scanning microscopy images of γ-H2AX immunostaining and mCherry expression with or without the administration of DSB inducer. Bars, 100 μm.

c. Quantification of γ-H2AX activation by imaging analysis. The data are expressed as means ± s.e.m. (n = 9). *$P < 0.05$ (Student's t-test). N.S., not significant.

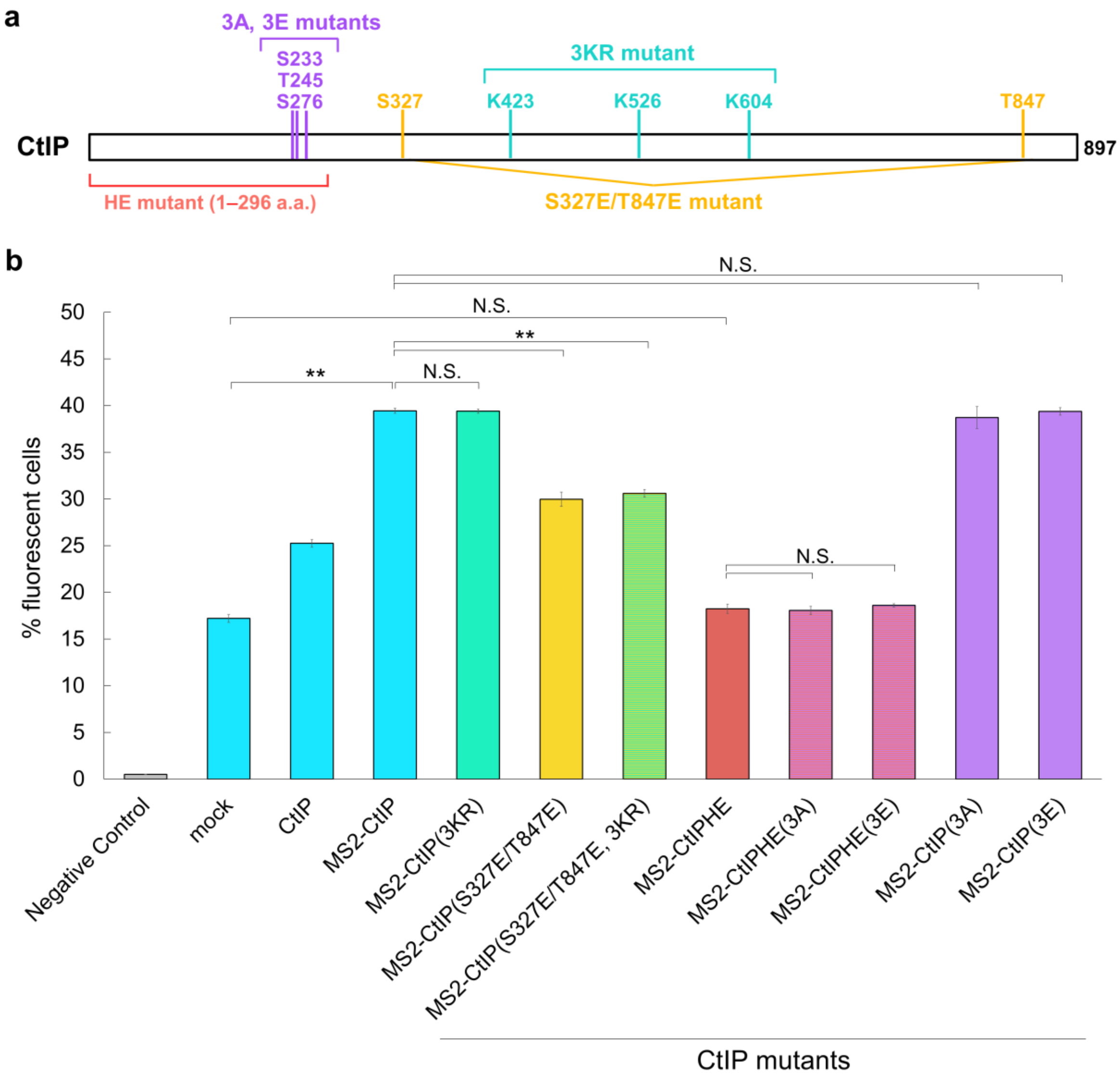


**Supplementary Figure 10. Characterization of MS2-CtIP mutants in the LoAD system.**

a. Schematic of CtIP mutants.

b. The knock-in-enhancing effect calculated by FACS analysis with or without MS2-CtIP mutants. Data are expressed as means ± s.e.m. (n = 3). **P < 0.01 (Student's t-test). N.S., not significant.

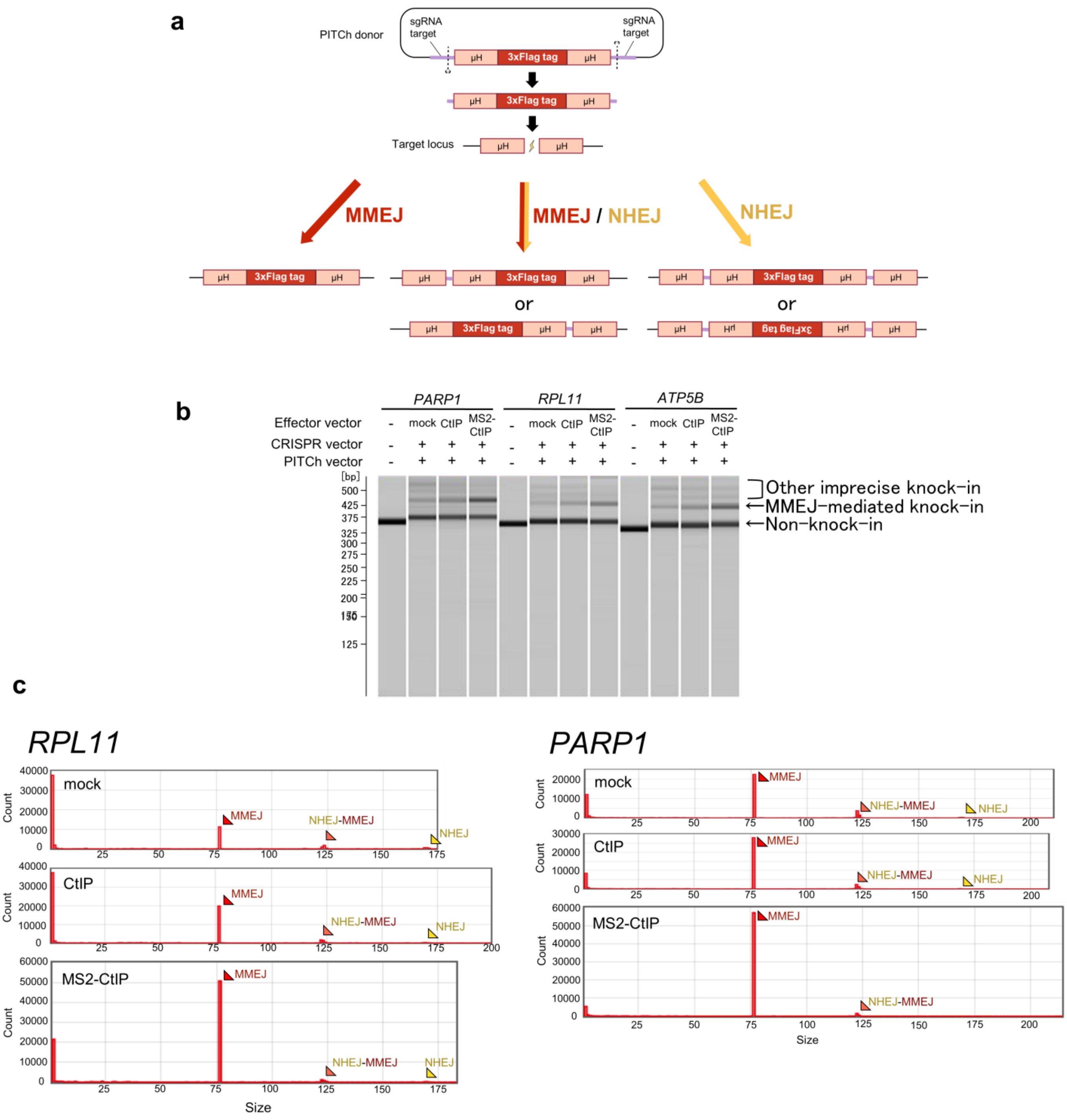


**Supplementary Figure 11. Analysis of 3× Flag knock-in outcomes, related to Figure 2**

a. Schematic of the MMEJ-, MMEJ/NHEJ-, and NHEJ-mediated knock-ins. µH, microhomology.

b. Pseudo-gel image of out-out PCR products. The densities of the bands with the expected sizes of MMEJ-mediated knock-in were highest in the samples transfected with MS2-CtIP, followed by CtIP and mock.
c. Histograms of the read counts at the *RPL11* and *PARP1* loci, separated by the lengths of inserts. MMEJ, MMEJ/NHEJ, and NHEJ show the expected sizes of the inserts mediated by MMEJ at both knock-in junctions, NHEJ at one junction and MMEJ at the other, and NHEJ at both junctions, respectively, as illustrated in Supplementary Figure 11a.

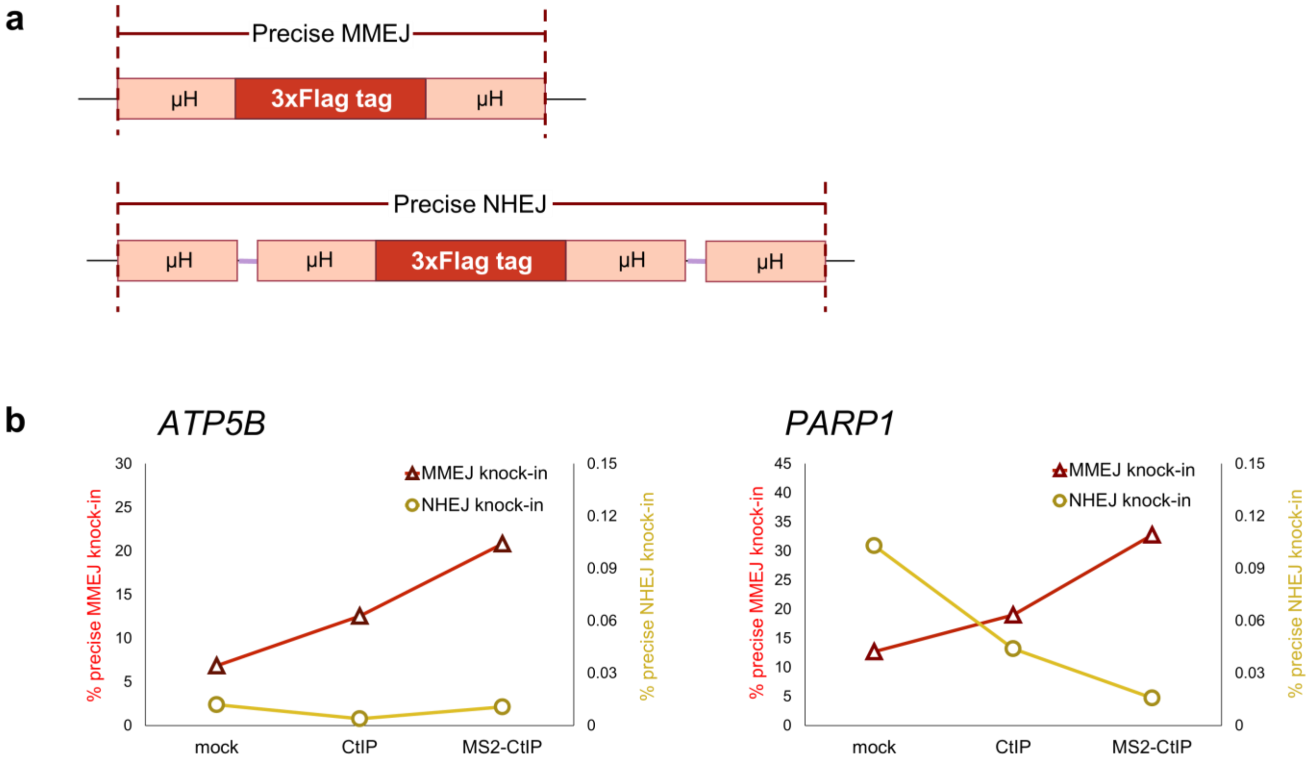


**Supplementary Figure 12. Analysis of the frequencies of precise MMEJ- and NHEJ-mediated knock-ins, related to Figure 2d**

a. Schematic of the precise MMEJ- and NHEJ-mediated knock-in alleles. Precise knock-ins were defined as illustrated.

b. Percentages of precise knock-in alleles mediated by MMEJ and NHEJ among total NGS reads at the *ATP5B* and *PARP1* loci.

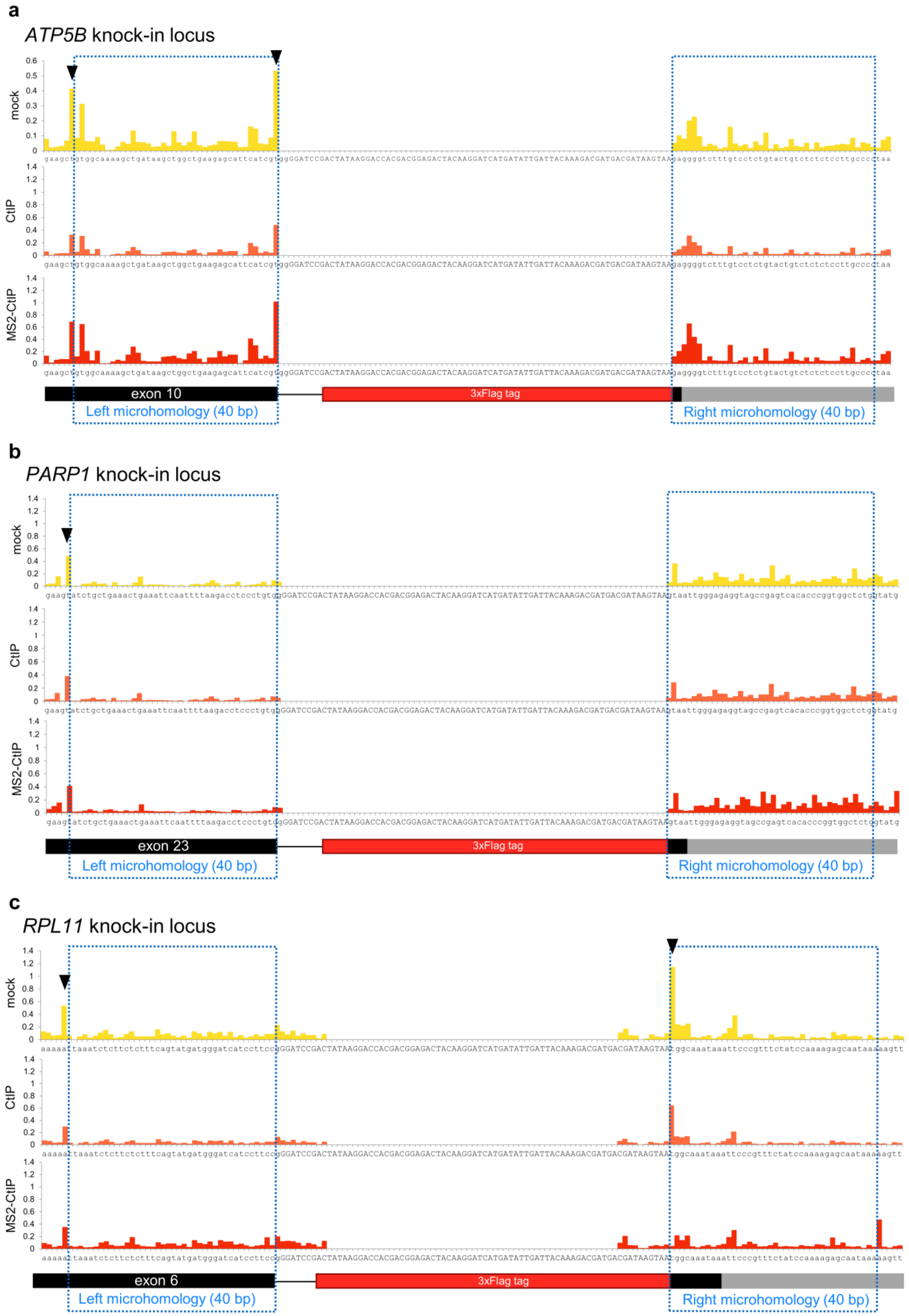
a
ATP5B knock-in locus
mock
CtIP
MS2-CtIP
exon 10
3xFlag tag
Left microhomology (40 bp)
Right microhomology (40 bp)
b
PARP1 knock-in locus
mock
CtIP
MS2-CtIP
exon 23
3xFlag tag
Left microhomology (40 bp)
Right microhomology (40 bp)
c
RPL11 knock-in locus
mock
CtIP
MS2-CtIP
exon 6
3xFlag tag
Left microhomology (40 bp)
Right microhomology (40 bp)

**Supplementary Figure 13. Profiles of base substitutions in and around microhomologies of 3× Flag knock-in samples**

The frequencies of base substitutions at the *ATP5B* (a), *PARP1* (b), and *RPL11* (c) knock-in loci are shown. Similar distributions were observed among mock, CtIP, and MS2-CtIP samples with some tendency of relatively high frequency of base substitutions around the ends of microhomologies (black triangles).

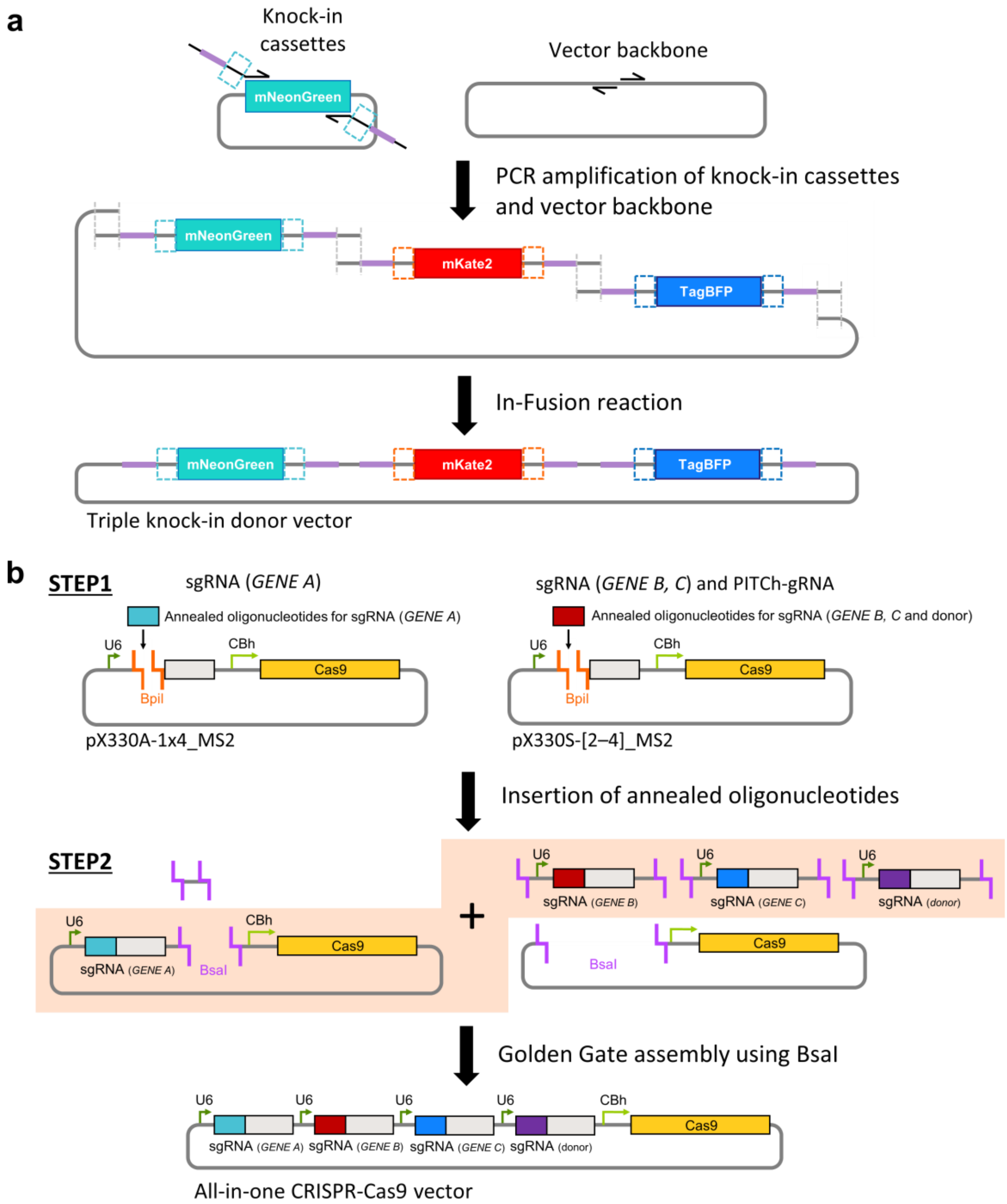


**Supplementary Figure 14. Schematic illustration of the construction methods of triple knock-in donor (a) and all-in-one CRISPR-Cas9 (b) vectors, related to Figure 3a**

The triple knock-in donor vector was constructed by one-step PCR and In-Fusion cloning. The all-in-one CRISPR-Cas9 vector was constructed by two-step cloning method.

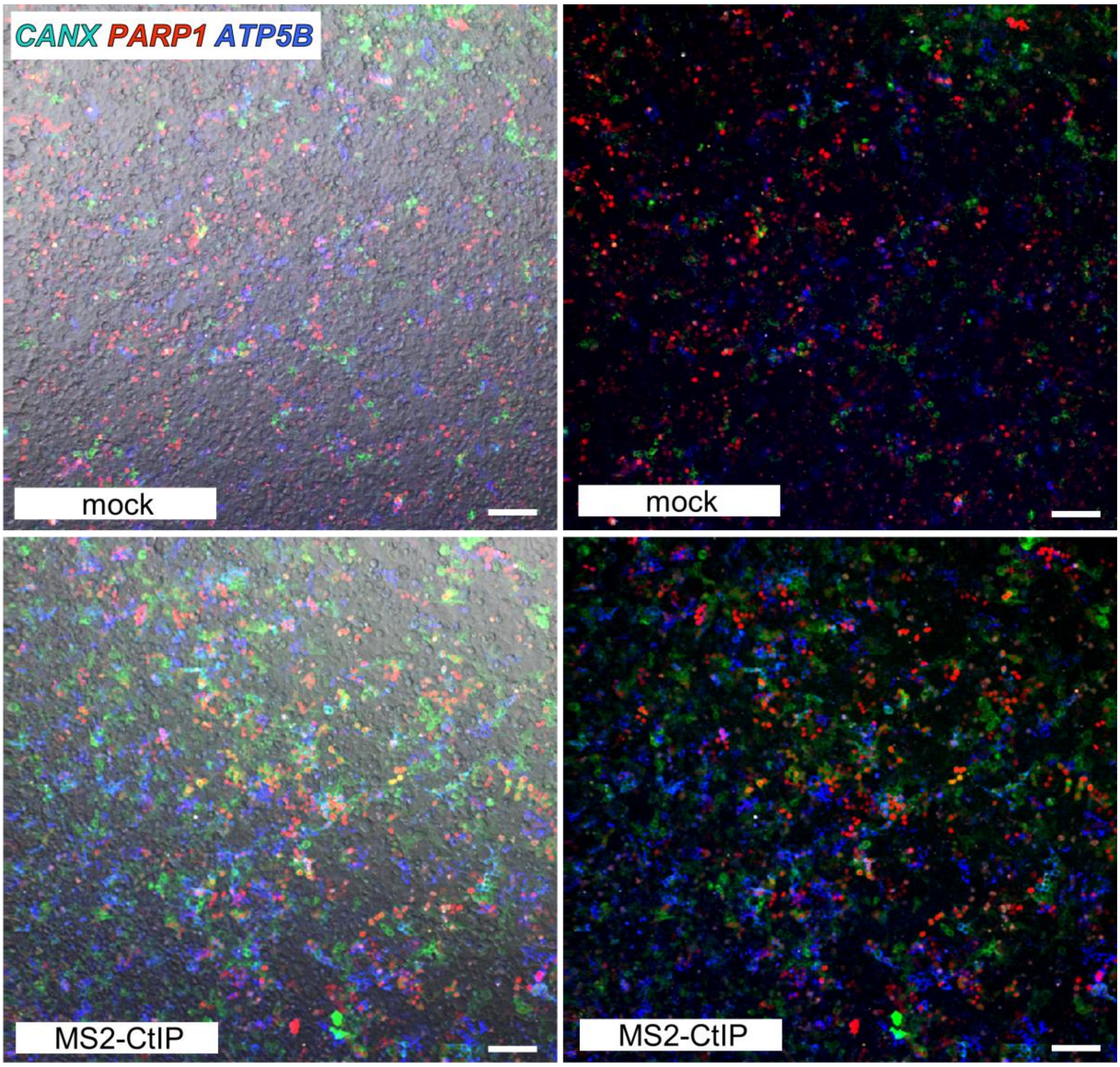


**Supplementary Figure 15. Knock-in-enhancing effect of MS2-CtIP LoADing in triple gene knock-in**

Merged images of green, red, and blue fluorescence with (left images) or without (right images) differential interference contrast, obtained with a laser-scanning microscope. Heterogeneous patterns of triple fluorescence were observed both in mock and in MS2-CtIP-LoADed cells, and a higher frequency of cells with fluorescence was detected in MS2-CtIP-LoADded cells than in mock cells. Bars, 100 μm.

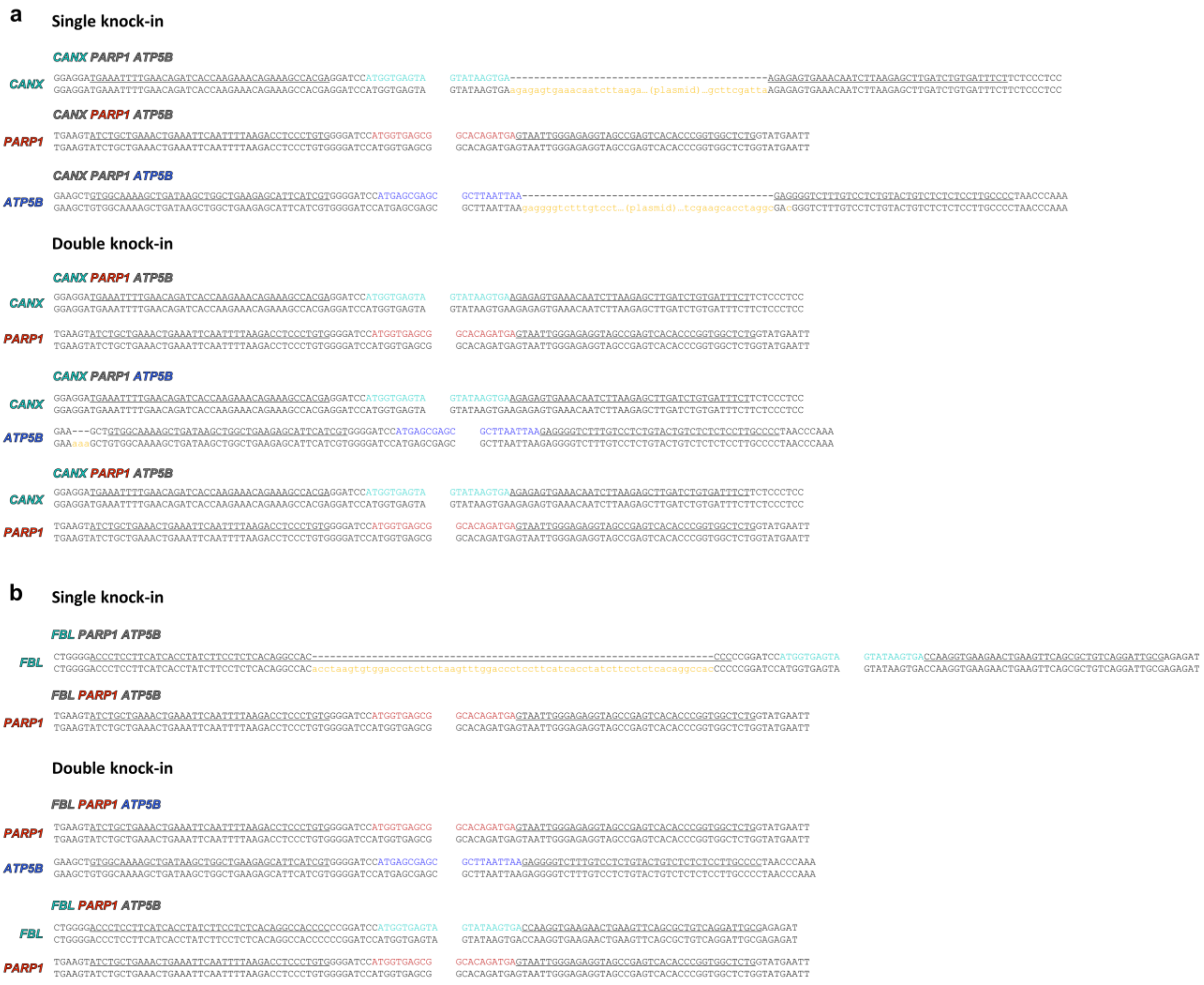


**Supplementary Figure 16. Sanger sequencing of knock-in junctions of single and double knock-in clones, related to Figure 3f**

The intended knock-in sequences are shown at the top of each sequence. Green, red, and blue letters indicate the coding sequences of mNeonGreen, mKate2, and TagBFP, respectively. Light blue letters indicate substitutions or insertions. Dashes indicate deletions. Underlines indicate microhomologies.

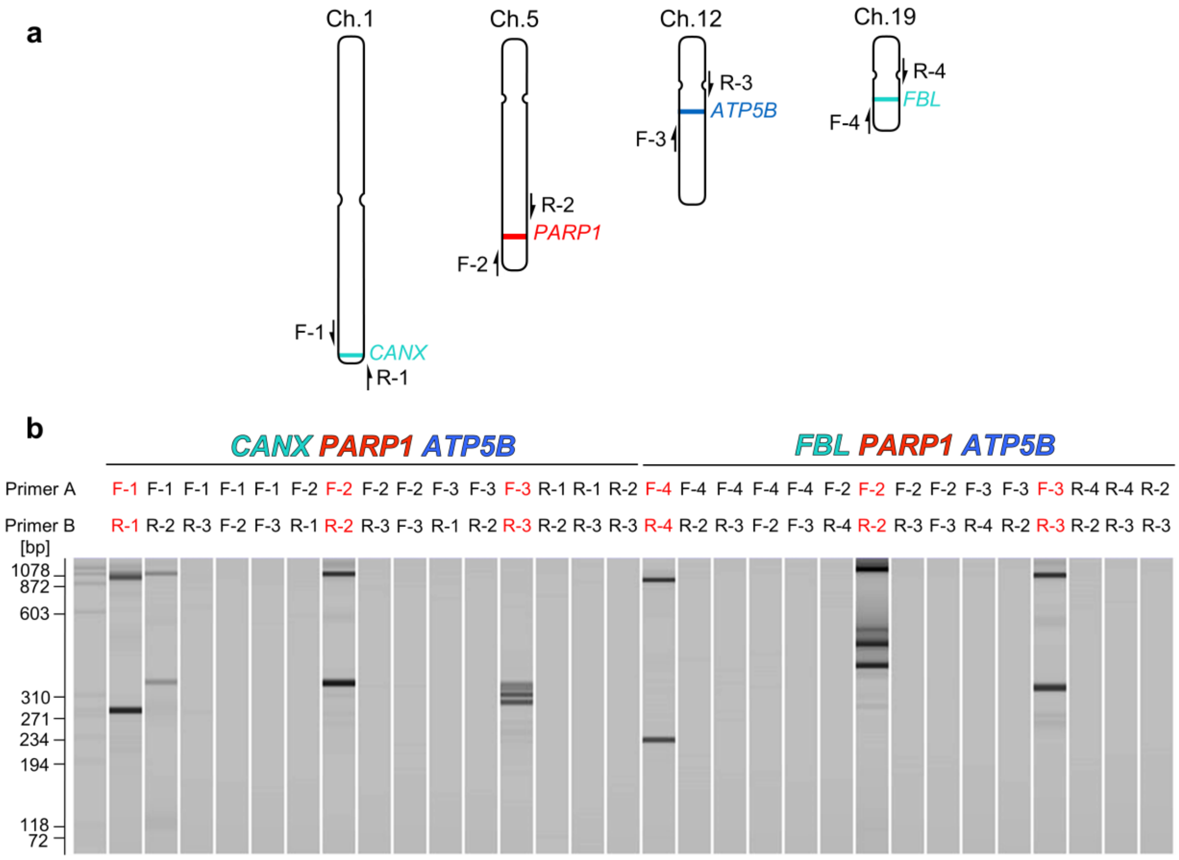


**Supplementary Figure 17. Translocation analysis of two triple knock-in clones**

a. Schematic of the targeted positions and designed primers.

b. PCR detection of translocation using all possible primer pairs. The primer pairs in red letters indicate untranslocated amplicons.

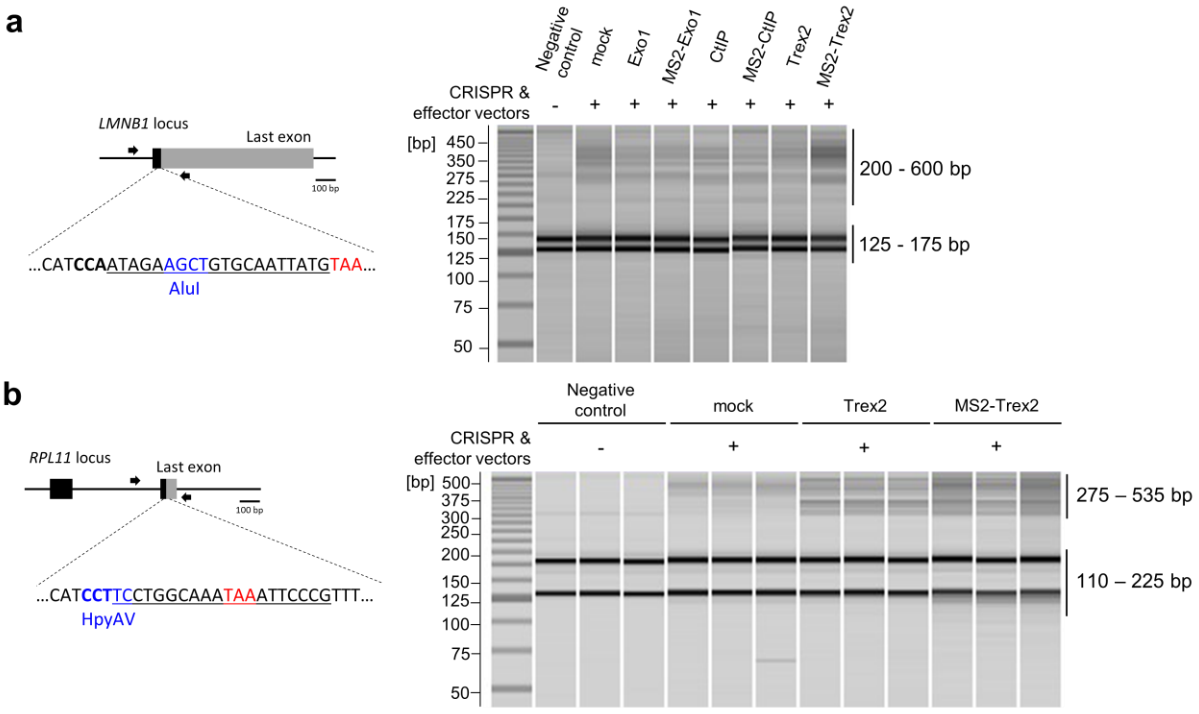


**c** **mock**

```
AAATCTCTTCTCTTTCAGTATGATGGGATCATCCTTCCTGGCAAATAAATTCCCGTTTCTATCCAAAAGAGCAATAAAAAGTTTTCA  WT
AAATCTCTTCTCTTTCAGTATGATGGGATCATCCTTCCTGGCAAATAAATTCCCGTTTCTATCCAAAAGAGCAATAAAAAGTTTTCA        x8 No mutation
AAATCTCTTCTCTTTCAGTATGATGGGATCATCC-------------------CGTTTCTATCCAAAAGAGCAATAAAAAGTTTTCA  -19   x1 Deletion
AAATCTCTTCTCTTTCAGTATGATGGGAT---------------------TCCCGTTTCTATCCAAAAGAGCAATAAAAAGTTTTCA  -21   x1 Deletion
AAATCTCTTCTCTTTCAGTATGATGGGATCATCCTTCCTtGGCAAATAAATTCCCGTTTCTATCCAAAAGAGCAATAAAAAGTTTTCA  +1   x5 Insertion
AAATCTCTTCTCTTTCAGTATGATGGGA--------atttatttg-------CCGTTTCTATCCAAAAGAGCAATAAAAGGTTTTCA  -24+9 x1 Mixed
```

**Trex2**

```
AAATCTCTTCTCTTTCAGTATGATGGGATCATCCTTCCTGGCAAATAAATTCCCGTTTCTATCCAAAAGAGCAATAAAAAGTTTTCA  WT
AAATCTCTTCTCTTTCAGTATGATGGGATCATCCTTCCTGGCAAATAAATTCCCGTTTCTATCCAAAAGAGCAATAAAAAGTTTTCA      x7 No mutation
AAATCTCTTCTCTTTCAGTATGATGGGATCATCCTTCCTG-CAAATAAATTCCCGTTTCTATCCAAAAGAGCAATAAAAAGTTTTCA  -1  x1 Deletion
AAATCTCTTCTCTTTCAGTATGATGGGATCATCCTTCC-GGCAAATAAATTCCCGTTTCTATCCAAAAGAGCAATAAAAAGTTTTCA  -1  x2 Deletion
AAATCTCTTCTCTTTCAGTATGATGGGATCATCCTTC-TGGCAAATAAATTCCCGTTTCTATCCAAAAGAGCAATAAAAAGTTTTCA  -1  x1 Deletion
AAATCTCTTCTCTTTCAGTATGATGGGATCATCCTTCCT---AAATAAATTCCCGTTTCTATCCAAAAGAGCAATAAAAAGTTTTCA  -3  x1 Deletion
AAATCTCTTCTCTTTCAGTATGATGGGATCATCCTTCCTG-----------CCCGTTTCTATCCAAAAGAGCAATAAAAAGTTTTCA  -11 x1 Deletion
AAATCTCTTCTCTTTCAGTATG----------------TGGCAAATAAATTCCCGTTTCTATCCAAAAGAGCAATAAAAAGTTTTCA  -16 x1 Deletion
AAATCTCT------------------------------------------------------CCAAAAGAGCAATAAAAAGTTTTCA  -54 x1 Deletion
AAATCTCTTCTCTTTCAGTATGATGGGATCATCCTTCCTtGGCAAATAAATTCCCGTTTCTATCCAAAAGAGCAATAAAAAGTTTTCA  +1 x3 Insertion
AAATCTCTTCTCTTTCAGTATGATGGGATCATCCTTCCTtGGCAAATAAATTCCCGTTTCTATCCAAAAGAGtAATAAAAAGTTTTCA  +1 x1 Insertion
```

**MS2-Trex2**

```
AAATCTCTTCTCTTTCAGTATGATGGGATCATCCTTCCTGGCAAATAAATTCCCGTTTCTATCCAAAAGAGCAATAAAAAGTTTTCA  WT
AAATCTCTTCTCTTTCAGTATGATGGGATCATCCTTCCTGGCAAATAAATTCCCGTTTCTATCCAAAAGAGCAATAAAAAGTTTTCA          x6 No mutation
AAATCTCTTCTCTTTCAGTATGATGGGATCATCCTTCC---CAAATAAATTCCCGTTTCTATCCAAAAGAGCAATAAAAAGTTTTCA  -3      x1 Deletion
AAATCTCTTCTCTTTCAGTATGATGGGATCATCCTTCCT----AATAAATTCCCGTTTCTATCCAAAAGAGCAATAAAAAGTTTTCA  -4      x1 Deletion
AAATCTCTTCTCTTTCAGTATGATGGGATCATCCTTCCT-------AAATTCCCGTTTCTATCCAAAAGAGCAATAAAAAGTTTTCA  -7      x1 Deletion
AAATCTCTTCTCTTTCAGTATGATGGGATCATCCTTCCT--------AATTCCCGTTTCTATCCAAAAGAGCAATAAAAAGTTTTCA  -8      x1 Deletion
AAATCTCTTCTCTTTCAGTATGATGGGATCA------------AATAAATTCCCGTTTCTATCCAAAAGAGCAATAAAAAGTTTTCA  -12     x1 Deletion
AAATCTCTTCTCTTTCAGTATGATGGGATCATCCTTCC---------------CGTTTCTATCCAAAAGAGCAATAAAAAGTTTTCA  -15     x1 Deletion
AAATCTCTTCTCTTTCAGTATGATGGGATCATCCTTCCTG-----------------------------AGCAATAAAAAGTTTTCA  -29     x1 Deletion
AAATCTCTTCTCTTTCAGTATGATGGGATCATCCTTCC------------------------------------------------A  -48     x1 Deletion
AAATCTCTTCTCTTTCAGTATGATGGGATCATCCTTCC---------------------(76 bp)---------------------  -76     x1 Deletion
---------------------------------------(211 bp)---------------------------------------  -211    x1 Deletion
...(plasmid)...accgccgccttctatgaaaggttgGGCAAATAAATTCCCGTTTCTATCCAAAAGAGCAATAAAAAGTTTTCA  -98+709 x1 Mixed
```

**Supplementary Figure 18. Analysis of mutations by RFLP and subcloned sequencing, related to Figure 4a–d**

a. Schematic of the RFLP analysis at the *LMNB1* locus (left panel) and pseudo-gel image of AluI-treated samples (right panel). Black box indicates coding sequence. Gray box indicates 3′ UTR. Black arrows indicate the positions of

primers. Underline indicates the target sequence of sgRNA. Bold letters indicate PAM sequence. Red letters indicate the stop codon.

b. Schematic of the RFLP analysis at the *RPL11* locus (left panel) and pseudo-gel image of HpyAV-treated samples (right panel). Black box indicates coding sequence. Gray box indicates 3′ UTR. Black arrows indicate the positions of primers. Underline indicates the target sequence of sgRNA. Bold letters indicate PAM sequence. Red letters indicate the stop codon.

c. Sequences and frequencies of bacterially cloned PCR products of the *RPL11* gene, related to Figure 4d. The wild-type sequence is shown at the top of each sequence. Red letters indicate substitutions or insertions. Dashes indicate deletions. Underlines indicate sgRNA target sequences. Bold letters indicate PAM sequences.

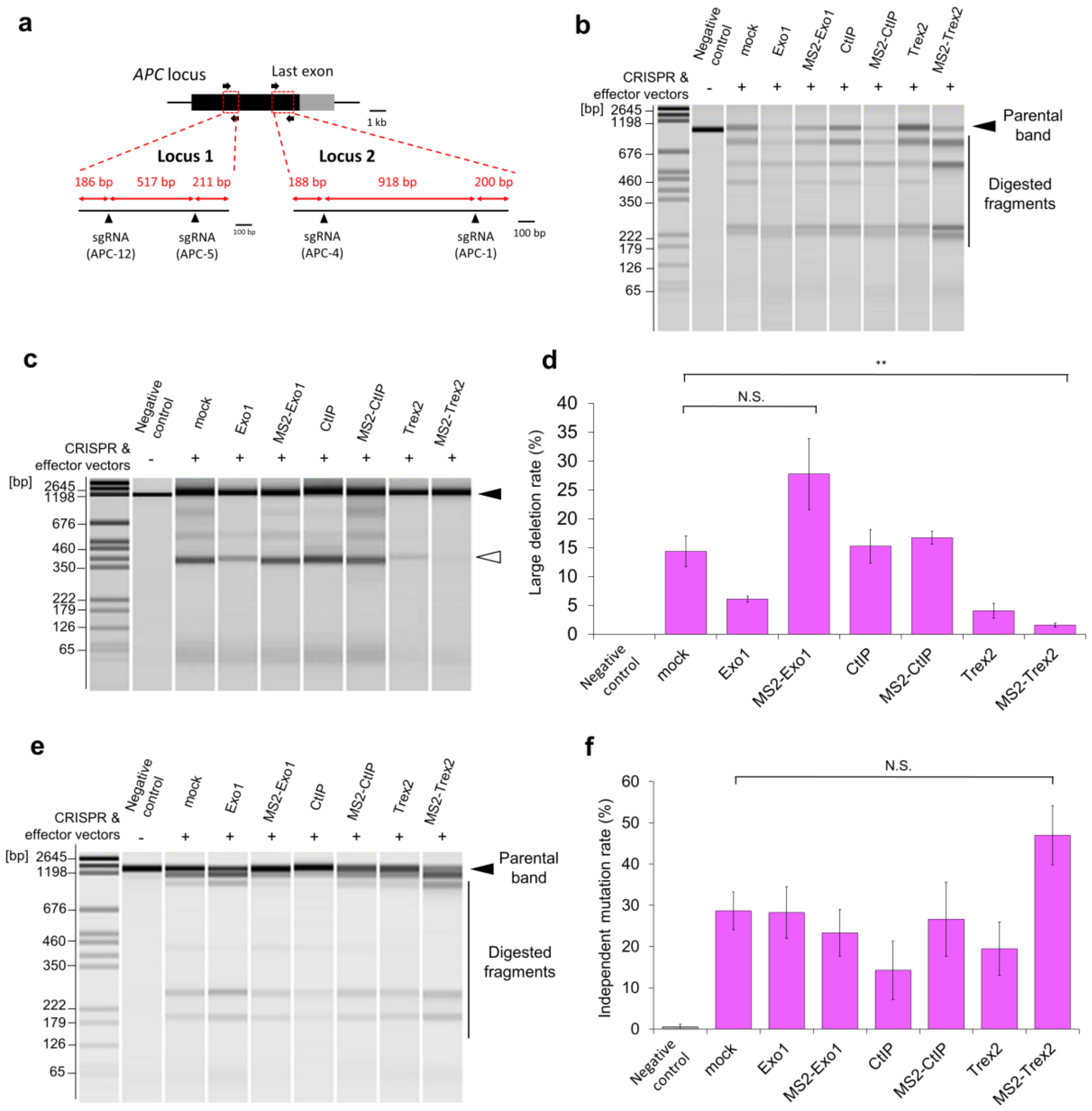


**Supplementary Figure 19. Analysis of chromosomal deletion and independent mutations, related to Figure 4e–h**

a. Schematic of the analysis. Two pairs of sgRNAs (APC-12 and APC-5, and APC-4 and APC-1) were used to analyze chromosomal deletion and independent mutations. Black box indicates coding sequence. Gray box indicates 3′ UTR. Black arrows indicate the positions of primers. Black arrowheads indicate the positions of sgRNA target sites.

b. Pseudo-gel image of the Cel-I analysis at *APC* locus 1, related to Figure 4h.

c. Pseudo-gel image of the out-out PCR products at *APC* locus 2. Black and white arrowheads indicate the expected positions of the wild-type and chromosomally deleted PCR products, respectively.
d. Percentages of chromosomally deleted alleles among all the out-out PCR products at *APC* locus 2. Data are expressed as means ± s.e.m. (n = 3). **P < 0.01 (Student's t-test). N.S., not significant.
e. Pseudo-gel image of the Cel-I analysis, quantitating the frequency of independently mutated alleles among the full-length PCR products indicated by the black arrowhead in Supplementary Figure 4c.
f. Percentages of independently mutated alleles quantitated by the Cel-I analysis, related to Supplementary Figure 4e. Data are expressed as means ± s.e.m. (n = 3). N.S., not significant.

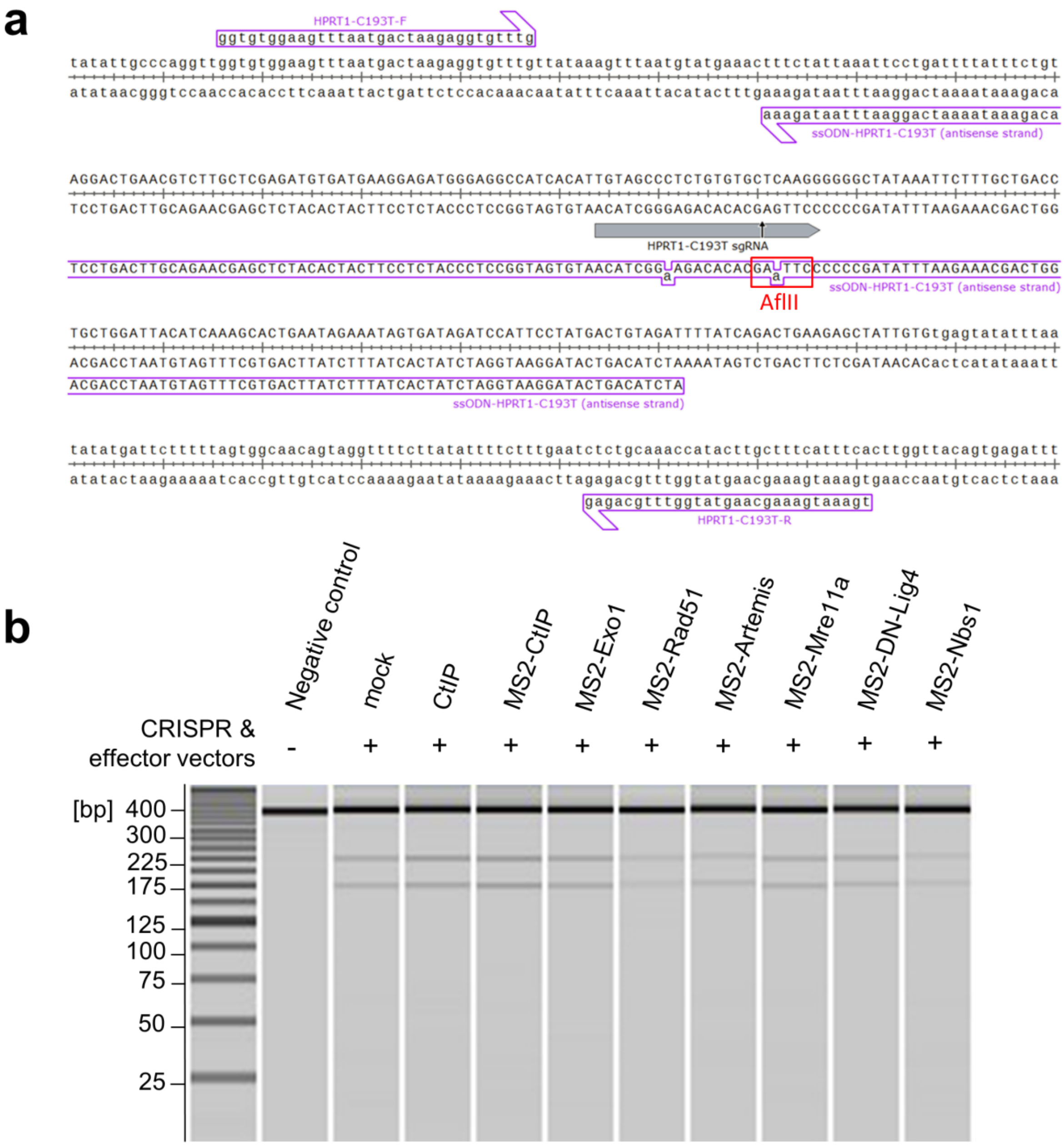


**Supplementary Figure 20. Analysis of SSTR-mediated knock-in, related to Figures 4i and j**

a. Schematic of SSTR-mediated knock-in, illustrated using the SnapGene Viewer software (http://www.snapgene.com/products/snapgene_viewer/), except the annotation of the AflII site.

b. Pseudo-gel image of the RFLP analysis, related to Figure 4j.

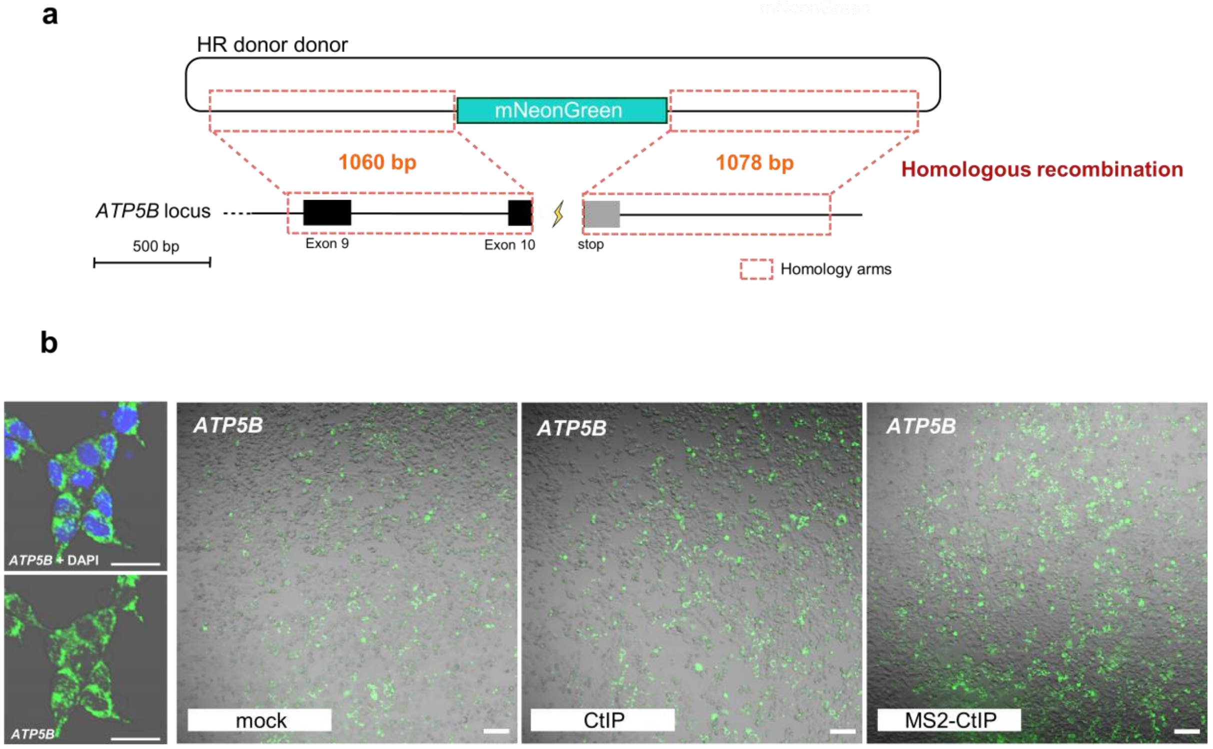


**Supplementary Figure 21. Analysis of HR-mediated knock-in, related to Figures 4k and l**

a. Schematic of the HR-mediated knock-in.
b. Fluorescence images of the HR-mediated knock-in cells, related to Figure 4l. Bars: left two images, 30 µm; right three images, 100 µm.
