# Supplementary material for: Biased genome editing using the local accumulation of DSB repair molecules system
Source: Nat Commun. 2018 Aug 16;9:3270. doi: 10.1038/s41467-018-05773-6 (PMC6095859; doi:10.1038/s41467-018-05773-6)
Supplement: Supplementary file 3 — Description of Additional Supplementary Files [file 41467_2018_5773_MOESM3_ESM.pdf]

Description of Additional Supplementary Files

File Name: Supplementary Data 1
Description: Synthesized oligonucleotides and DNA fragments used in this study.

File Name: Supplementary Data 2
Description: The NGS data analysed by CRISPResso software.

File Name: Supplementary Data 3
Description: The NGS data analysed by Cas-Analyzer software.

File Name: Supplementary Data 4
Description: The plasmid sequences of CtIP vector, MS2-CtIP vector, and the PITCh donor vectors used in Figures 2d–f.
